# KLK4 Induces Anti-Tumor Effects in Human Xenograft Mouse Models of Orthotopic and Metastatic Prostate Cancer

**DOI:** 10.3390/cancers12123501

**Published:** 2020-11-24

**Authors:** Brian W.-C. Tse, Thomas Kryza, Mei-Chun Yeh, Ying Dong, Kamil A. Sokolowski, Carina Walpole, Tobias Dreyer, Johanna Felber, Jonathan Harris, Viktor Magdolen, Pamela J. Russell, Judith A. Clements

**Affiliations:** 1 Preclinical Imaging Facility, Translational Research Institute, Brisbane 4102, Australia; kamil.sokolowski@tri.edu.au; 2 Australian Prostate Cancer Research Centre—Queensland, Institute of Health and Biomedical Innovation, Translational Research Institute, Queensland University of Technology, Brisbane 4102, Australia; thomas.kryza@mater.uq.edu.au (T.K.); mei.yeh@uq.edu.au (M.-C.Y.); y.dong@qut.edu.au (Y.D.); carina.walpole@mater.uq.edu.au (C.W.); pamela.russell@qut.edu.au (P.J.R.); j.clements@qut.edu.au (J.A.C.); 3 School of Biomedical Sciences, Faculty of Health, Queensland University of Technology, Brisbane 4102, Australia; j2.harris@qut.edu.au; 4 Translational Research Institute, Mater Research Institute—The University of Queensland, Brisbane 4102, Australia; 5 Clinical Research Unit, Department of Obstetrics and Gynecology, Technical University of Munich, 81675 Munich, Germany; Tobi.3er@gmx.de (T.D.); johannafelber@gmx.de (J.F.); viktor.magdolen@tum.de (V.M.)

**Keywords:** kallikrein-related peptidase 4, KLK4, prostate cancer, metastasis, tumor xenografts, imaging

## Abstract

**Simple Summary:**

The serine protease kallikrein-related peptidase 4 (KLK4) has been reported to potentially play a role in the progression of prostate cancer and other cancer types. However, most of these reports have been limited to in vitro studies. In vivo cancer models offer greater complexity to mimic the characteristics of cancer growth and metastasis in humans. In this study, we used in vivo models of prostate cancer and demonstrated that KLK4 can strongly inhibit the growth of primary prostate tumors as well as bone metastases. To our knowledge, this is the first report of an anti-tumor effect of KLK4 in prostate cancer in vivo.

**Abstract:**

Recent reports have suggested the role of kallikrein-related peptidase 4 (KLK4) to be that of remodeling the tumor microenvironment in many cancers, including prostate cancer. Notably, these studies have suggested a pro-tumorigenic role for KLK4, especially in prostate cancer. However, these have been primarily in vitro studies, with limited in vivo studies performed to date. Herein, we employed an orthotopic inoculation xenograft model to mimic the growth of primary tumors, and an intracardiac injection to induce metastatic dissemination to determine the in vivo tumorigenic effects of KLK4 overexpressed in PC3 prostate cancer cells. Notably, we found that these KLK4-expressing cells gave rise to smaller localized tumors and decreased metastases than the parent PC-3 cells. To our knowledge, this is the first report of an anti-tumorigenic effect of KLK4, particularly in prostate cancer. These findings also provide a cautionary tale of the need for in vivo analyses to substantiate in vitro experimental data.

## 1. Introduction

Cancer growth and metastasis are driven not only by intrinsic cancer cell characteristics but also by their interaction with the tumor microenvironment [1]. Complex interactions with the surrounding extracellular matrix, stromal fibroblasts, blood vessels, and cytokines profoundly affect cancer cell behavior. While in vitro cancer models are useful to shed light on biochemical pathways, they often do not provide sufficient complexity to adequately study these interactions. In vivo cancer models offer greater complexity to mimic characteristics of cancer growth and metastasis in humans.

Kallikrein-related peptidase 4 (KLK4), a trypsin-like serine protease which is secreted by epithelial cells into their microenvironment [2,3,4], has been reported to have paradoxical effects in prostate cancer (PCa). However, to date, these functional studies of KLK4 in cancer have been limited to in vitro models. We previously reported that KLK4 induces cancer-associated fibroblast features in prostate-derived stromal cells [5], induces epithelial to mesenchymal transition (EMT)-like characteristics [6], and activates matrix metalloproteinase-1 (MMP-1) [7], which, together, could impact tumor growth and metastasis. Of note, KLK4-transfected PC3 cells had increased migration towards osteoblast-like SaOs2 cell conditioned medium and greater attachment to the bone-matrix proteins, collagens I and IV [8]. However, we have also shown that KLK4-overexpressing cells exhibit a slower proliferative rate, suggesting that KLK4 may have tumor limiting properties [6]. A major knowledge gap concerns how KLK4 secretion by PCa cells influences their in vivo growth and dissemination patterns. 

For this study, PC3 PCa cells, which do not naturally express KLK4, were selected due to their bone metastasis origin and high aggressiveness, thereby modeling advanced prostate cancer, and importantly, propensity to develop primary tumors and bone metastases in mice, thus providing a model to test our earlier in vitro findings as described above [5,6,7,8]. PCa progression was modeled by injection of PC3 cells, modified to over-express human full length KLK4, in immune-deficient mice via two routes: orthotopically in the prostate to mimic the growth of primary tumors, and intracardiac (left ventricular) to induce metastatic dissemination. In vivo tumor growth patterns were assessed using the imaging modalities, optical (bioluminescence), microPET-CT, photoacoustics, and high-resolution microCT (ex vivo). We also used a second cell line, prostate specific membrane antigen (PSMA)-expressing PC3 cells, so that the tumor burden could be assessed using a clinically utilized imaging technique PSMA PET-CT.

To the best of our knowledge, this is the first report of the phenomenon that KLK4 over-expression induces anti-tumor effects in vivo, particularly in PCa. We demonstrate using complex human xenograft mouse models of orthotopic and metastatic PCa that, contrary to previous reports, KLK4 expression in advanced disease does not correlate with an increased aggressiveness. This is critical as it demonstrates that targeting KLK4 might not be rational in advanced disease. Moreover, it also highlights the importance of using in vivo analyses to substantiate in vitro experimental data.

## 2. Results

### 2.1. Low Kallikrein-Related Peptidase 4 (KLK4) mRNA Levels Are Associated with Gleason Grade and Metastatic Lesions

**Figure 1:**
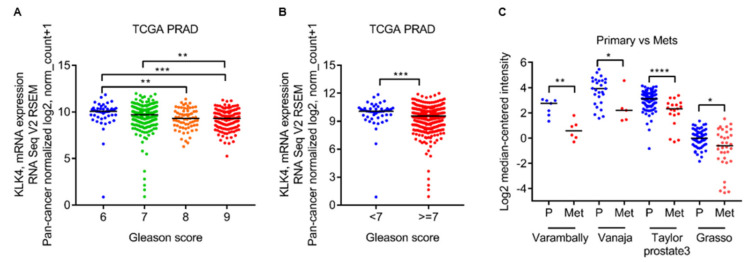
Transcriptomic analysis of kallikrein-related peptidase 4 (KLK4) expression in clinical prostate cancer specimens. Scatter plots showing KLK4 mRNA levels in clinical samples of (**A**) Gleason 6, 7, 8 versus 9, (**B**) Gleason <7 versus ≥7, and (**C**) primary versus metastasis across multiple data sets. Log2 median centered gene expression data were obtained from Oncomine; horizontal lines indicate median value. Statistical analysis for (**A**) was Kruskal-Wallis test followed by Dunn’s multiple comparison’s test, and for (**B**) and (**C**) was Mann-Whitney test. * *p* < 0.05, ** *p* < 0.01, *** *p* < 0.001, **** *p* < 0.0001.

To shed light on a potential role for KLK4 in prostate cancer progression, we examined KLK4 mRNA levels in several large clinical prostate cancer cohorts in publically available transcriptomic datasets. Analysis of RNA sequencing data from The Cancer Genome Atlas (TCGA) Prostate Adenocarcinoma (PRAD) cohort revealed that KLK4 mRNA levels were significantly lower in specimens of Gleason grade 8 (*n* = 67) or 9 (*n* = 142) compared to grade 6 (*n* = 50) (Figure 1A), and Gleason grade >7 had lower KLK4 transcript levels than Gleason grade <7 (Figure 1B) [9]. Gene profiling through RNA microarray analysis of primary tumors and metastatic samples by Varambally et al. [10], Vanaja et al. [11], Taylor et al. [12], and Grasso et al. [13] all revealed that KLK4 levels were significantly lower in metastases (Figure 1C). Collectively, this clinical evidence correlates low KLK4 levels with prostate cancer progression.

### 2.2. KLK4-Transfected PC3-PSMA Cells Secrete Enzymatically Active KLK4 Protein, and Exhibit Reduced Proliferative Rate In Vitro

**Figure 2:**
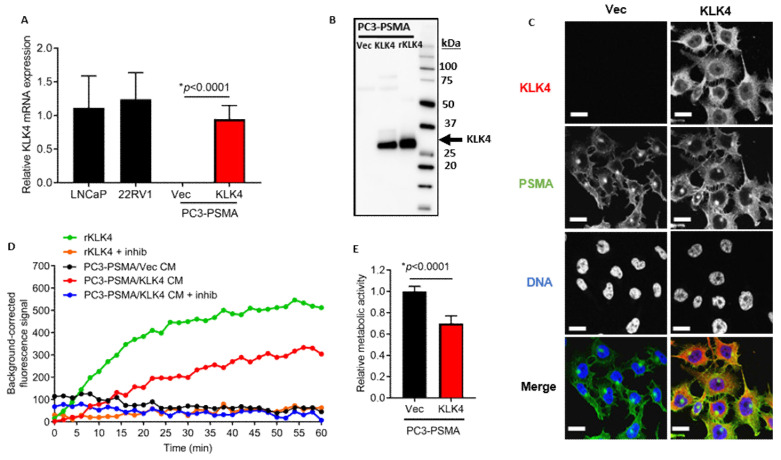
KLK4-transfected PC3-PSMA cells secrete KLK4 protein that is enzymatically active and exhibit reduced proliferation rate. (**A**) Relative KLK4 mRNA expression in LNCaP, 22RV1, PC3-PSMA/Vector and /KLK4 cells. Statistical test comparing PC3-PSMA/Vector and /KLK4 cells was unpaired T test. (**B**) Western blot analysis for KLK4 in conditioned media (CM) of transfected cells, with recombinant (r) KLK4 as positive control. (**C**) Merged expression of KLK4 (red) and PSMA (green) protein by immunofluorescence in cell lines; DAPI staining (blue; DNA)—bottom panel. Upper panels give single images in grey scale. Scale bar indicates 20 µm. (**D**) Enzyme activity assay with transfected concentrated CM or recombinant KLK4, with or without inhibitor (SFTI-FCQR, 4 µM). (**E**) Cell proliferation rate comparison performed with Alamar Blue reagent 48 h after cell seeding; unpaired *t* test for statistical analysis (*n* = 3 independent experiments).

To enable investigation on the effects of KLK4 on prostate cancer behavior in vitro and in vivo, PC3-PSMA cells, which do not express KLK4, were modified to over-express KLK4. QPCR analysis revealed that PC3-PSMA/KLK4 cells express similar levels of KLK4 mRNA as LNCaP and 22RV1 cells (Figure 2A), which express endogenous KLK4 transcripts. Western blotting detected KLK4 protein in PC3-PSMA/KLK4 cell CM with a single ~30 kDa band (Figure 2B and Figure S5) indicating that the transfected KLK4 was secreted. Immunofluorescence microscopy revealed strong cytoplasmic staining for KLK4 protein within PC3-PSMA/KLK4 cells (Figure 2C). Enzymatic activity was detectable in PC3-PSMA/KLK4 cell CM, which was inhibited by co-incubation with SFTI-FCQR, a selective KLK4 active site inhibitor (Figure 2D). No KLK4 transcript, protein, or enzymatic activity within CM was detectable with the control cell line PC3-PSMA/Vec. PC3-PSMA/KLK4 cells, as assessed using Alamar Blue reagent for metabolic activity, exhibited reduced in vitro proliferation rate (Figure 2E) as we had reported previously [6]. Similar results were observed in our characterization of the KLK4-transfected parent PC3 cell counterparts generated in parallel (Figures S1 and S5).

### 2.3. Tumor Secretion of KLK4 Inhibits the Growth of Orthotopically Implanted Prostate Tumors in Mice

**Figure 3:**
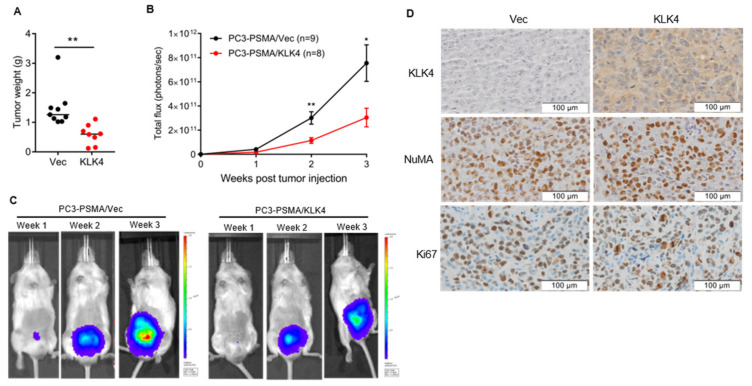
KLK4 production by PC3-PSMA cells inhibits in vivo growth of orthotopic tumors. (**A**) Scatter plot of post-mortem weights of orthotopic PC3-PSMA/Vec and /KLK4 tumors at week 3; horizontal line indicates median value. Statistical analysis was Mann-Whitney test. (**B**) Mean tumor bioluminescence ± SEM for each group at three time points; statistics by unpaired T-test. (**C**) Representative images of tumor bioluminescence in mice bearing PC3-PSMA/Vec or /KLK4 tumors. (**D**) Immunohistochemical staining of orthotopic tumors for KLK4, NuMA, and Ki67. All presented Figures are from pooled data from two independent in vivo experiments. * *p* < 0.05, ** *p* < 0.01.

To investigate the effects of KLK4 on the growth of primary tumors, NOD-SCID mice were injected in the prostate with PC3-PSMA/KLK4 or PC3-PSMA/Vec cells. Three weeks post-tumor implantation, mice were euthanized and the post-necropsy weight of PC3-PSMA/KLK4 tumors was significantly lower than PC3-PSMA/Vec tumors (Figure 3A). The KLK4 tumor inhibitory effects was confirmed by weekly in vivo optical imaging, with the bioluminescence of KLK4-secreting tumors observed lower than control tumors over the three weeks (Figure 3B,C). Both PC3-PSMA/Vec and /KLK4 cells had the same baseline bioluminescence, as demonstrated by in vitro imaging of the cell lines prior to injection (Figure S2), confirming the difference in in vivo tumor bioluminescence is indeed due to a difference in tumor burden. Immunohistochemical analysis of resected tumors showed strong staining for KLK4 in PC3-PSMA/KLK4 tumors, confirming the secretion of large amounts of KLK4 in vivo (Figure 3D). The tumors were also confirmed to be of human origin through positive staining for human nuclear mitotic apparatus protein (NuMA). For both KLK4 and control groups, the majority of tumor areas stained positively for the proliferation marker Ki67, with no difference in staining level between groups indicating that there are still proliferating cells in the KLK4 tumors albeit limited growth overall (Figure 3D). To examine how tumor over-expression of KLK4 modulates the prostatic microenvironment, photoacoustic imaging for oxygen saturation was performed, but no differences were observed in tumors between the groups (Figure S3). PC3-KLK4 tumors showed a trend towards being smaller than PC3-Vec tumors, although considerable variation was noted (Figure S4A). However, the bioluminescence signals from PC3-KLK4 tumors were significantly lower than in the PC3-Vec group at weeks 3 and 4 (Figure S4B), which is consistent with data from the PC3-PSMA-KLK4 model (Figure 3B).

### 2.4. Tumor Secretion of KLK4 Is Associated with Significantly Lower Metastatic Tumor Burden in Mice

**Figure 4:**
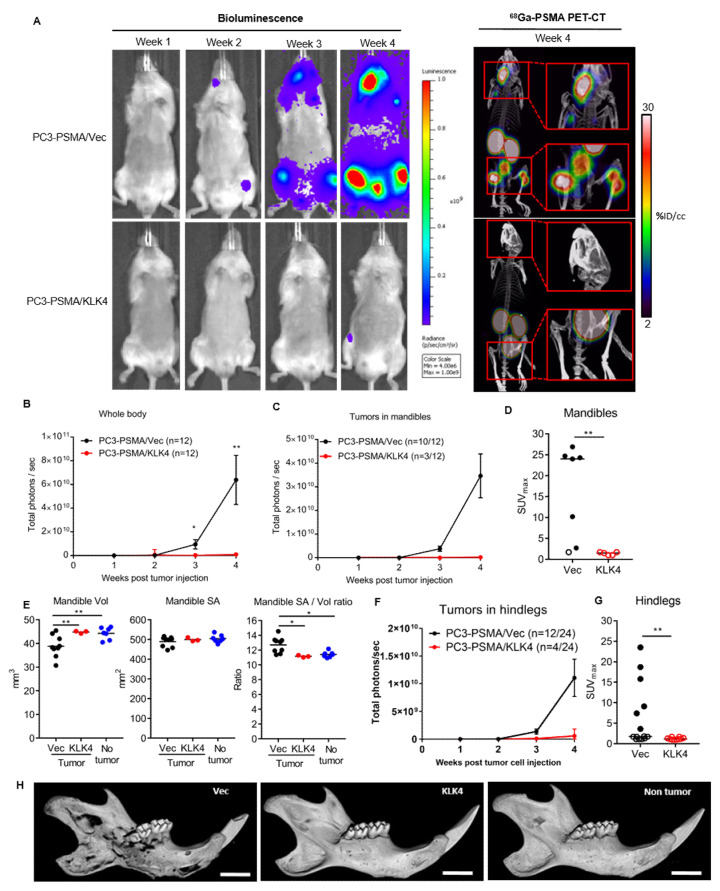
KLK4 production by PC3-PSMA cells induces potent anti-metastatic effects. (**A**) Representative images of tumor bioluminescence (weeks 1–4) and PET-CT imaging with Ga68-PSMA (week 4; endpoint) of mice injected intracardiac with PC3-PSMA/Vec or /KLK4 tumor cells. Mean tumor bioluminescence ± SEM from region of interest (ROI) drawn over the (**B**) whole body, (**C**) mandible, and (**F**) hindlegs, of mice over 4 weeks. Statistical analysis performed was un-paired T-test. PET-CT SUV_max_ values from ROIs drawn over (**D**) mandible and (**G**) hind legs (closed circles, tumors present; open circle, no tumor/background). Horizontal line indicates median value; statistical analysis was Mann-Whitney test. (**E**) Scatter plot of bone volume (vol), surface area (SA), or SA/Vol ratio, values of mandibles from mice bearing tumors following ex vivo microCT imaging. One way ANOVA followed by Tukey’s multiple comparison test. (**H**) Representative images of mandibles from each group. All presented Figures are from pooled data from two in vivo independent experiments. * *p* < 0.05, ** *p* < 0.01.; scale bar indicates 3mm.

Since KLK4 tumor production inhibited the growth of orthotopically implanted tumors, we next investigated if similar effects were also observed in a bone-metastastic model whereby tumor cells were injected intracardiac in mice for arterial blood dissemination. We utilized two independent imaging modalities, namely bioluminescence and PET-CT imaging with the clinically utilized radiotracer ^68^Ga-PSMA-HBED-CC, to cross-validate tumor burden observations. Mice injected intracardiac with PC3-PSMA/Vec cells developed multiple pseudo-metastases throughout the body, with the most common anatomical sites being the mandible and hind legs (Figure 4A). In stark contrast, mice injected intracardiac with PC3-PSMA/KLK4 cells developed significantly less tumors and had much lower whole-body bioluminescence over the four weeks (Figure 4A,B). The incidence of mandible tumors at four weeks was 25% (3/12) for mice injected with PC3-PMSA/KLK4 cells, in contrast to 83% (10/12) for PC3-PMSA/Vec tumor cells (Figure 4C). The mandible tumors that did develop in the KLK4 group were also significantly smaller based on bioluminescence and PET-CT imaging (SUV_max_ values) (Figure 4C,D). We also examined the degree of tumor-induced bone lysis on the mandibles by ex vivo microCT imaging. The bone surface area to volume ratio was the highest for mandibles harboring PC3-PSMA/Vec tumors, as compared to those harboring PC3-PSMA/KLK4 tumors, or non-tumor controls (Figure 4E,H). Thus, KLK4 not only reduced the incidence and size of mandible tumors but also limited the degree of tumor-induced bone lysis. The anti-tumor effects associated with KLK4 were also observed in the hind legs, where 16% (four of 24, from twelve mice) of hind legs from mice injected with PC3-PSMA/KLK4 had detectable tumors, in contrast to 50% (12 of 24) with the PC3-PSMA/Vec group (Figure 4F). Tumors that did develop in the hind legs from the KLK4 group were also significantly smaller (Figure 4F,G). However, in the classical PC3 model, there was no difference in tumor burden in mice injected with PC3-KLK4 or PC3-Vec tumors (Figure S4C).

## 3. Discussion

The work reported in this manuscript is, to our knowledge, the first to use an in vivo model in order to investigate the functional role of the secreted form of KLK4 in advanced PCa. Our results demonstrate that, in the context of advanced disease, the secreted KLK4 protease exerts an anti-tumorigenic effect reducing intra-prostatic growth and metastasis formation of the aggressive, bone metastatic PC3 cells (Figure 3 and Figure 4).

This finding perhaps is reflected by the variation in *KLK4* expression levels during PCa progression obtained from various transcriptomic datasets showing a decreasing expression with progression of prostate tumors as well as a lower expression in PCa-metastasis compared to primary PCa (Figure 1). The elevated expression of *KLK4/*KLK4 in PCa tissues compared to normal prostate or benign tumors early in disease is validated by several studies [5,14,15,16,17], but the correlation between its level of expression and clinical parameters in PCa progression has given contradictory results. In concordance with the transcriptomic data sets, Seiz et al. [17] reported lower protein expression of KLK4 in T3+4 compared to T1+2 stage disease, whereas two other studies reported increased *KLK4*/KLK4 in patients with higher T stage and higher Gleason score [14,16]. Whether this reflects cohort differences, heterogeneity of tissues, splice variants, relatively small cohorts used in these studies (<60 patients) or differences in KLK4 antibody specificity is yet to be established.

The precise involvement of KLK4 in PCa is not clearly determined with a combination of studies identifying multiple molecular mechanisms regulated by this protein and which can, depending on the context, be interpreted as pro- or anti-tumorigenic events. For example, we have shown that expression of the KLK4 protease could induce an EMT-like phenotype in PC3 PCa cells (mesenchymal phenotype, a reduction in proliferation rate and an increase in cell migration) which is a cellular phenotype often associated with more aggressive disease but also known to lead to a reduction of the primary tumor burden in favor of the formation of metastasis [6,18]. Several studies have also demonstrated that the KLK4 protease could regulate proteinase-activated receptors (PARs, [5,19,20]) expressed by both PCa cells (including PC3 cells) [21] and stromal cells found in the PCa microenvironment resulting in pro-tumorigenic effects. These included the activation of mitogen-activated protein kinase (MAPK) in cancer cells through PAR-2 activation [19] and the induction of cancer-associated fibroblast (CAF)-like features in stromal cells through PAR-1 activation which can play an essential role during PCa initiation [5,20]. Although CAFs are usually considered as exerting pro-tumorigenic functions, recent reports indicate that their role is far more complex and that they can also play anti-tumorigenic functions through their role in the remodeling of ECM and in the eliciting of an antitumor immune response [22,23]. Additionally, the KLK4 protease has been suggested as a potential regulator of the interaction between PCa cells and the microenvironment of metastatic tumors. It was shown that KLK4 expression is increased in PCa cells in the presence of osteoblast-like cells and that the expression of KLK4 in PCa cells facilitated their migration toward osteoblast-like cells [8]. Notably, all of these studies were performed in vitro, and extrapolated to represent disease progression in vivo, although both the in vivo local microenvironment and that of metastatic dissemination are far more complex, multifactorial situations.

The related KLK family member KLK3, well known as prostate specific antigen (PSA), has been reported also to have pro- and anti-tumorigenic effects. Its role in the tumor microenvironment is still not fully understood in the context of PCa progression [3,24]. For instance, although PSA can promote tumor activity by activation of insulin growth factor (IGF) through degradation of IGF binding proteins, and degradation of matrix proteins such as laminin, it is also known to have anti-angiogenic effects [2,3]. Another pathway that both PSA and KLK4 have been suggested to regulate is the transforming growth factor (TGF)β pathway which is known to have both pro- and anti-tumor effects [25,26]. PSA can activate the latent/pro-form of TGFβ [27,28] and KLK4 can activate/degrade several proteins associated with TGFβ activation, such as thrombospondin 1, particularly in the PC3 cell microenvironment [7]. Indeed, TGFβ related proteins have been shown to be key regulators of PC3 cell proliferation [29,30] with a concomitant increase in plasminogen activator inhibitor-1 (PAI-1) [29], a known serpin inhibitor of KLK4 which could act as a further control on KLK4 activity [16,31]. In the same light, we have previously shown that, in the presence of osteoblast-like cells, PCa cell-derived KLK4 binds to a high molecular weight binding protein, possibly a serpin, thus inactivating KLK4 [8]. Activation of the TGFβ pathway was also observed in a proteomic analysis of the secretome of an ovarian cancer cell line engineered to overexpress KLK4, KLK5, KLK6 and KLK7 [32]. Regulation of TGFβ-1 activity by KLK4 is also a key event in dentine enamel matrix maturation [33]. Thus, although KLK4 has been clearly shown to interact with the TGFβ-1 pathway, its precise role, and whether it effects a pro- or anti-tumorigenic action, is still not known.

Like PSA, KLK4 expression is regulated by androgens and the androgen receptor (AR) pathway [2], which is an essential signaling pathway during development and progression of PCa. Although this pathway could not contribute to KLK4 regulation on this occasion given that the AR negative PC3 cell line was used for these studies, it is interesting to note that KLK4 can regulate the bioavailability of circulating androgens through its capacity to degrade sex hormone-binding globulin (SHBG) which is a key protein carrier of androgens [34]. This degradation of SHBG is predicted to be a negative regulator of the AR-pathway as it would result in a decrease in androgen stability. Whether this would contribute to an overall dampening of any androgen regulated events in the mouse tumor microenvironment leading to tumor suppression, is not known. Notably, in this study, we chose to focus on more advanced/bone metastatic disease when the tumor becomes resistant to AR effects, hence the choice of the PC3 cell line which also does not have endogenous KLK4 expression.

These discrepancies between studies investigating the function of KLK4 in prostate tumors can be attributed likely to the diverse methodologies employed. However, there are also other forms of KLK4 which exist in the tumor microenvironment and for which there is no known function. Since the discovery of the KLK4 gene it has been demonstrated that multiple KLK4 transcripts variants are produced in cancer cells [35,36]. Recently, we showed that at least five different KLK4 transcripts are expressed in prostate cancer tissues with the reference transcript encoding the classical KLK4 protease (pre-pro-KLK4) not being the predominantly expressed form of KLK4 [37]. This is in line with previous reports that identified KLK4 to be a cytoplasmic/nuclear protein in prostate tissues [35,38,39,40]. However, a recent proteomic study was able to identify the classical form of the KLK4 protease in seminal plasma and blood serum from individuals with confirmed prostate cancer and negative biopsy without being able to identify any significant difference between the two sample groups, but thus confirming KLK4 is secreted from PCa as well as the normal prostate [41]. Other earlier, albeit smaller, studies also demonstrated KLK4 secretion into seminal fluid with the suggestion of higher levels in healthy prostate tissues but could not demonstrate major differences in benign compared to malignant prostate tissues [4,42]. These different transcriptomic, proteomic, and functional studies show that, in fact, several forms of KLK4 may coexist in PCa tissues and that they could exert very different effects.

In this study, the use of complex orthotopic and metastatic PCa models, with advanced imaging techniques, provided a unique opportunity to examine the effects of KLK4 in PCa progression. While over-expression models are useful to determine the function of genes/proteins, a limitation is that the scenario is somewhat artificial. However, since there are currently no cell line models of advanced PCa available which display a high expression of KLK4, it is difficult to employ gene suppression or inhibition models. Moreover, few PCa cell lines grow when implanted orthotopically in the prostate, or that they establish bone metastases when injected into the arterial blood circulation (intracardiac), thus further limiting the repertoire of PCa models for in vivo use. Given that a key focus of our study was to determine the impact in the bone metastatic environment, the bone metastatic PC-3 cell line, which is also not androgen responsive (a feature of advanced PCa) and readily forms tumors in vivo, was selected and modified to over-express KLK4.

The finding that KLK4 exhibits anti-tumor effects in vivo has significant translational impact. Firstly, it suggests that targeting KLK4 might not be rational for advanced disease. Secondly, and more importantly, it will also open the way for future investigations to understand the mechanisms underlying the negative impact of KLK4 expression/activity on advanced PCa tumor growth and metastasis formation in order to identify novel possible targets.

With the experimental models used herein, we cannot comment on the potential function of KLK4 during the first steps of metastasis such as migration from the primary tumor and invasion of blood vessels nor the influence of KLK4 on the androgen axis, or vice versa, in prostate cancer. More complex in vivo studies, perhaps utilizing co-injection of (androgen sensitive and/or insensitive) PCa cells with human CAFs, and /or fully humanized mice. Such models would enable greater interaction of proteins of human origin, and thereby more closely mimic the human situation. That notwithstanding, our work demonstrates that the classical form of KLK4 (secreted serine protease) exerts an anti-tumor role in both an orthotopic and experimental metastasis in vivo model using the aggressive PC3 PCa bone metastatic-derived cell line.

## 4. Materials and Methods

### 4.1. Evaluation of KLK4 Expression in Clinical Samples

Normalized KLK4 expression data from Publicly available microarray (Oncomine/www.oncomine.org) and The Cancer Genome Atlas (TCGA)/University of California Santa Cruz Xenabrowser (https://xenabrowser.net/) gene (count+1) expression datasets were used.

### 4.2. Cell Culture

The PSMA-expressing PC3 cell line, PC3-PIP (referred to as “PC3-PSMA”), and the classical PC3 cell line (referred to as “PC3”) were obtained from Dr Warren Heston (Cleveland Clinic, Cleveland, OH, USA), and American Type Culture Collection (ATCC; Manassas, UA, USA), respectively. Both cell lines were previously modified to express DsRed protein and luciferase as described [43]. LNCaP and 22RV1 cells were also sourced from the ATCC. All cell lines were cultured in phenol red-free RPMI media containing 5% fetal bovine serum (ThermoFisher Scientific, Brisbane, Australia), and incubated at 37 °C in humidified atmosphere of 5% CO_2_/air. Cells were authenticated by short tandem repeat (STR) profiling (March 2018), and regularly tested for mycoplasma infection.

### 4.3. Generation of KLK4 Transfected Cell Lines

PC3-PSMA and PC3 cells were modified to express wild type pre-pro-KLK4 (uniprot ID: Q9Y5K2-1), resulting in PC3-PSMA/KLK4 and PC3/KLK4 cells, respectively. Briefly, KLK4 full length cDNA was cloned into the mammalian expression plasmid pRc/RSV (Thermo Fisher Scientific, Brisbane, Australia) followed by verification of sequence as described [44]. Cells transfected with the empty vector were used as controls (PC3-PSMA/Vec, and PC3-Vec). Briefly, 2 µg of plasmid DNA was transfected into cells using Lipofectamine 2000 reagent (Thermo Fisher Scientific) as per the manufacturer’s instructions, and stable transfectants were selected using G418 antibiotic at 500 µg/mL for 3 weeks.

### 4.4. Quantitative PCR

qPCR was performed as described [45]. The primer sequences were as follows: *KLK4*, forward 5′-GAGGGCAAGACCAGAAGGACT-3′, and reverse 5′-TTTCCGAAAGACACAAGGCC-3′ (allows detection of KLK4 transcript variants-1 (classical structure) and -2); *RPL32* (reference gene), forward 5′-GCACCAGTCAGACCGATATG-3′, and reverse 5′-ACTGGGCAGCATGTGCTTTG-3′.

### 4.5. Western Blotting

Serum-free conditioned media (CM) were concentrated 10-fold using Vivaspin500 3 kDa cut-off spin columns (Sartorius, Goettingen, Germany). 20 µL of 10-fold concentrated CM, or in-house produced recombinant KLK4 [5,7], were then mixed with 4X Bolt LDA sample buffer, heated at 95 °C, loaded into 4–12% NuPage Novex Bis-Tris Midi Gels (Thermo Fisher Scientific), run in 2-(*N*-morpholino)ethanesulfonic acid (MES) buffer, transferred to nitrocellulose membranes (Bio-Rad, Hercules, CA, USA) using the Transblot Turbo system (Bio-Rad, Hercules, CA, USA), then blocked in 5% skim milk in PBS containing 0.5% Tween 20 (PBS/T20). The primary antibody used was in house-produced #581 rabbit anti-human KLK4 [17] followed by PBS/T20 washes and goat-anti-rabbit IgG-HRP (Abcam, Cambridge, UK) secondary antibody. Antigen was detected using ECL detection kit (Millipore, Burlington, MA, USA) and imaged with ChemiDoc (Bio-Rad, Hercules, CA, USA).

### 4.6. Enzyme Activity Assay

To determine the enzymatic activity in serum-free CM of cells, 10-fold concentrated CM was incubated with the selective KLK4 active site inhibitor, sunflower trypsin inhibitor (SFTI-FCQR; 4 µM) [46], or PBS, at 37 °C for 30 min before fluorescence substrate addition (50 µM D-Val-Leu-Arg-7-amido-4-trifluoromethyl coumarin or VLR-AFC; Sigma-Aldrich, St Louis, MO, USA) in PBS. The initial rate of KLK4 activity (Δrelative fluorescence units/min; ΔRFU/min) was measured in a PolarStar Optima microplate reader (BMG Labtech, Offenburg, Germany) at ex 400 nm, em 505 nm, 37 °C.

### 4.7. Fluorescence Microscopy

Breifly, 5 × 10^4^ cells were seeded onto 12-mm diameter glass coverslips (Menzel-Glaser, Braunscheig, Germany) placed inside 24-well plates, cultured in growth media for 72 h, fixed in 4% paraformaldehyde, permeabilised in 0.5% Triton X100, and then blocked in 1% BSA/PBS. Primary antibodies used were: #581 rabbit anti-human KLK4, and #J591 mouse anti-human PSMA from Dr Neil Bander (Weill Medical Centre, Cornell University, Ithaca, NY, USA) [47]. Secondary antibodies used were AlexaFluor 750 goat-anti-rabbit IgG, and AlexaFluor 647 goat-and-mouse IgG (both Thermo Fisher Scientific). DAPI (Thermo Fisher Scientific) staining was performed for nuclear localisation. Cells were washed with PBS in between each of the aforementioned steps. Coverslips were mounted onto microscopic slides with Mowiol 4-88 (Sigma-Aldrich, St Louis, MO, USA) and fluorescence images were acquired with an Olympus FV1200 confocal microscope (Olympus, Tokyo, Japan).

### 4.8. Cell Proliferation Assay

Cells were seeded at 5000 cells/well in growth media in 96-well plates. After 48 h, 20 µL of Alamar Blue reagent (Thermo Fisher Scientific) was added per well and absorbance at 570 nm was measured using a FLUOstar Omega plate reader (BMG Labtech, Offenburg, Germany) after 2 h incubation at 37 °C.

### 4.9. Animal Ethics Statement

All studies were approved by the Animal Ethics Committees of The University of Queensland (AEC number: 542/15) and Queensland University of Technology, and conducted in accordance with the Australian Code for the Care and Use of Animals for Scientific Purposes.

### 4.10. Mice and Housing Conditions

Male immune-compromised NOD.CB17-prkd^SCID^ (NOD-SCID) mice, 6–7 weeks old, were sourced from the Australian Resources Centre (ARC; Perth, Australia). All mice were maintained at the Biological Resources Facility (a specific pathogen-free facility) at the Translational Research Institute. Mice were randomized into groups of five, housed in individual ventilated cages (IVCs; Techniplast, West Chester, PA, USA), at 22 °C with a 12 h light-dark cycle, fed with standard chow and water ad libitum.

### 4.11. In Vivo Tumorigenesis Studies

Cultured cells were detached from culture flasks with trypsin, washed and resuspended in PBS. Viability was assessed by trypan blue exclusion. In the orthotopic tumor model, 10^6^ cells/25 µL were injected into the ventral prostate of mice as described previously [48]. In the metastasis model, 2 × 10^5^ tumor cells in 100 µL were slowly injected into the left ventricle of mice for arterial blood dissemination, a technical procedure guided by a small animal ultrasound imaging station (Vevo 2100, Visualsonics, Toronto, Canada) as described [49]. All mice had health monitoring performed daily, with body weights recorded 2–3 times a week. At experimental endpoints (defined time points; see Figure Legends of individual experiments), mice were euthanised by carbon dioxide asphyxiation (rate of 20% chamber volume/min) followed by cervical dislocation.

### 4.12. Tumor Bioluminescence Imaging

Tumor development was monitored by weekly bioluminescence imaging using an IVIS Spectrum (Perkin Elmer, Waltham, MA, USA) as described [48]. Bioluminescence was analyzed using Living Imagine software 4.5.4 (Perkin Elmer). The total flux in photons/second (p/s) within each defined region of interest (ROI) provides a surrogate of tumor burden. For in vitro imaging, bioluminescent cells were seeded at 50,000 cells/well down to 50 cells/well (2-fold serial dilution) in 96-well plates. D-luciferin (Perkin Elmer) was added to each well (final concentration was 150 µg/mL of media) minutes prior to imaging.

### 4.13. PET-CT Imaging

The radiotracer ^68^Ga-PSMA HBED-CC was produced by Q-TRaCE, Department of Nuclear Medicine, Royal Brisbane and Women’s Hospital, Australia. Mice were injected with 5 MBq of ^68^Ga-PSMA HBED-CC diluted in saline via the tail vein, then 1 h later anaesthetised by isoflurane and placed inside an Inveon scanner (Siemens, Munich, Germany) for sequential PET and CT imaging. The imaging acquisition parameters used were as described [50]. PET activity per voxel was converted to bq/cc using a conversion factor obtained by scanning a cylindrical phantom filled with a known activity of ^68^Ga to account for PET scanner efficiency. Activity concentrations within tissue ROIs were expressed as percentage of the decay-corrected injected activity per cubic cm of tissue (% ID/cc; SUV) using Inveon Research Workplace software v4.2 (IRW; Siemens, Munich, Germany).

### 4.14. High Resolution microCT (Ex Vivo)

High resolution microCT imaging was performed using a Skyscan 1272 (Bruker, Billerica, MA, USA). Mouse skull specimens were fixed in 10% neutral-buffered formalin for 48 h, stored in 70% ethanol, then wrapped in moist tissue paper and transferred into 5 mL cylindrical plastic tubes for imaging. The scanning parameters were: 60 kV X ray voltage, 153 µA current, 625 ms exposure time, 14 µm isotropic voxel size, 0.6° rotation step (360° imaging), 3 frame averaging, 4 × 4 binning, and 0.5 mm Al filter. The datasets were reconstructed with NRecon (version 1.7.3.1; Bruker, Billerica, MA, USA) and InstaRecon (version 2.0.4.2; University of Illinois, Champaign, IL, USA) software using cone beam reconstruction (Feldkamp) algorithm and the following corrections applied: ring artefact reduction, smoothing, beam hardening, and post-alignment. CT analysis was performed using CTan software version (Bruker, Billerica, MA, USA), with the threshold for bone set at 1657 Hounsfield Units, and 3D visualizations of mandibles generated using CTVox software (Bruker, Billerica, MA, USA).

### 4.15. Immunohistochemical Staining of Xenograft Tumors

Resected tumors were fixed in 4% paraformaldehyde and paraffin embedded. Antigen retrieval was performed on 4 µm sections using sodium citrate buffer (10 mM, 0.05% Tween20, pH 6.0), endogenous peroxidase activity quenched with 3% (*v*/*v*) H_2_O_2_, followed by blocking with TBS-Tween 20/5% BSA. Immunohistochemical staining was performed using primary antibodies to KLK4 (#581), Ki67 (Cell Signalling Technology, Danvers, MA, USA) and NuMA (Abcam). Antigen visualisation was achieved using the Duo-EnVisionTM peroxidase (anti-rabbit and mouse) polymer detection system and 3,3’-diaminobenzidine (DAB; Agilent, Santa Clara, CA, USA). Sections were counterstained (Harris’ haematoxylin) and images viewed using an automated Olympus slide scanner (VS120) and associated software OlyVia (Olympus Life Sciences, Tokyo, Japan).

### 4.16. Statistical Analysis

All statistical analysis was performed using Graphpad Prism v8 software (Graphpad Software Inc., San Diego, CA, USA). See Figure legends for statistical test performed; * *p* < 0.05, ** *p* < 0.01, *** *p* < 0.001, **** *p* < 0.0001.

## 5. Conclusions

In conclusion, these reports show that the role of KLK4 proteins in PCa is very context- and, possibly, isoform-dependent and that proteins encoded by KLK4 could perform either pro- and/or anti-tumor functions during PCa progression. This finding has major implications for the development of KLK4 antagonists or agonists as therapeutics for cancer. However, additional work is required to determine the precise function of KLK4 during PCa tumorigenesis in both early and late stage/metastatic disease.

## Supplementary Materials

The following are available online at https://www.mdpi.com/2072-6694/12/12/3501/s1. Figure S1: In vitro characterization of classical “PC3” cells modified to express KLK4, Figure S2: In vitro bioluminescence imaging of cell lines. Figure S3. Photoacoustic imaging for oxygen saturation of orthotopically implanted PC3-PSMA/Vec or /KLK4 tumors at week 3 (endpoint), Figure S4: In vivo characterization of PC3-Vec and PC3-KLK4 cells injected intraprostatic and intracardiac in mice. Figure S5: Uncropped Western Blot image.
