# Supplementary material for: KLK4 Induces Anti-Tumor Effects in Human Xenograft Mouse Models of Orthotopic and Metastatic Prostate Cancer
Source: Cancers (Basel). 2020 Nov 24;12(12):3501. doi: 10.3390/cancers12123501 (PMC7761350; doi:10.3390/cancers12123501)
Supplement: Supplementary file 1 [file cancers-12-03501-s001.pdf]

SupplementaryMaterials

KLK4 Induces Anti-Tumor Effects in Human Xenograft Mouse Models of Orthotopic and Metastatic Prostate Cancer

Brian W-C Tse, Thomas Kryza, Mei-Chun Yeh, Ying Dong, Kamil A Sokolowski, Carina Walpole, Tobias Dreyer, Johanna Felber, Jonathan Harris, Viktor Magdolen, Pamela J Russell and Judith A Clements

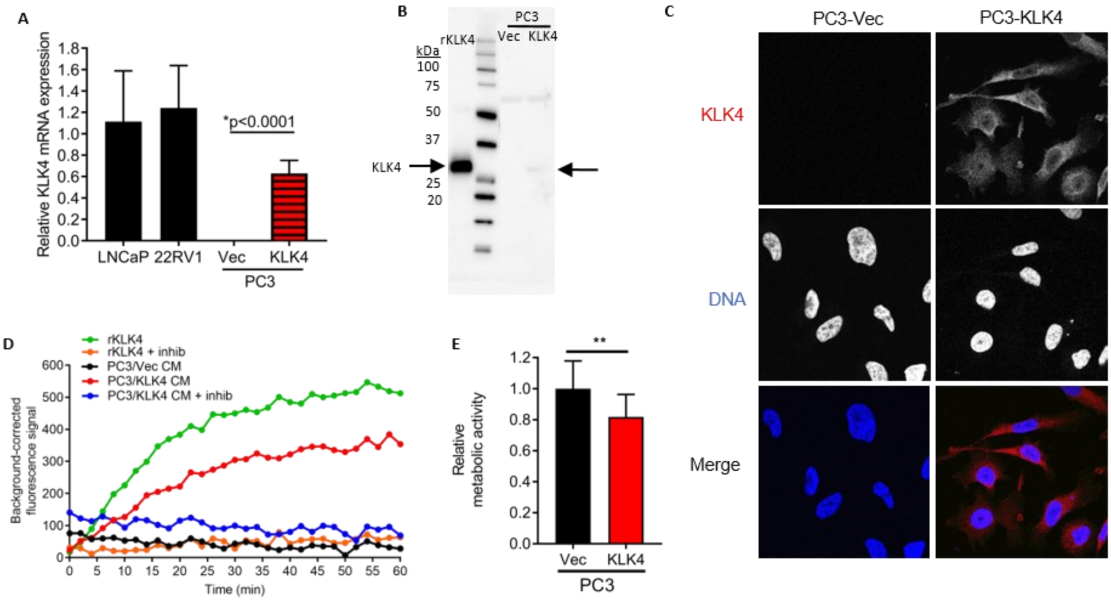

Figure S1. In vitro characterisation of classical "PC3" cells modified to express KLK4. **A**) Relative KLK4 mRNA expression in LNCaP, 22RV1, PC3-Vec and -KLK4 cells. Statistical test comparing PC3-Vec and -KLK4 cells was unpaired T test. **B**) Western blot analysis for KLK4 in conditioned media (CM) of transfected cells, with recombinant (r) KLK4 as positive control (cropped blot). **C**) Expression of KLK4 (red) protein by immunofluorescence in cell lines. DAPI (blue; DNA). **D**) Enzyme activity assay with transfect conditioned media or rKLK4, with or without inhibitor (SFTI-FCQR; 4µM). **E**) Cell proliferation rate comparison following 48 h incubation of cells with Alamar Blue reagent; unpaired T test for statistical analysis.

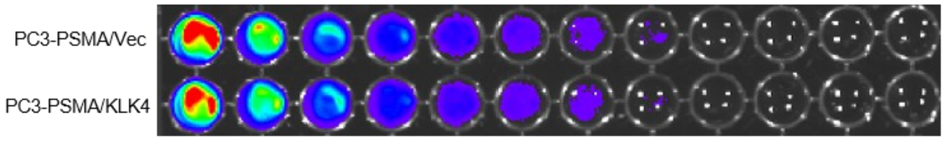

Figure S2. In vitro bioluminescence imaging of cell lines. Both PC3-PSMA/Vec and /KLK4 cells have the same baseline bioluminescence level. 50 000 cells were seeded in the wells (first column) then diluted 2-fold in subsequent wells.

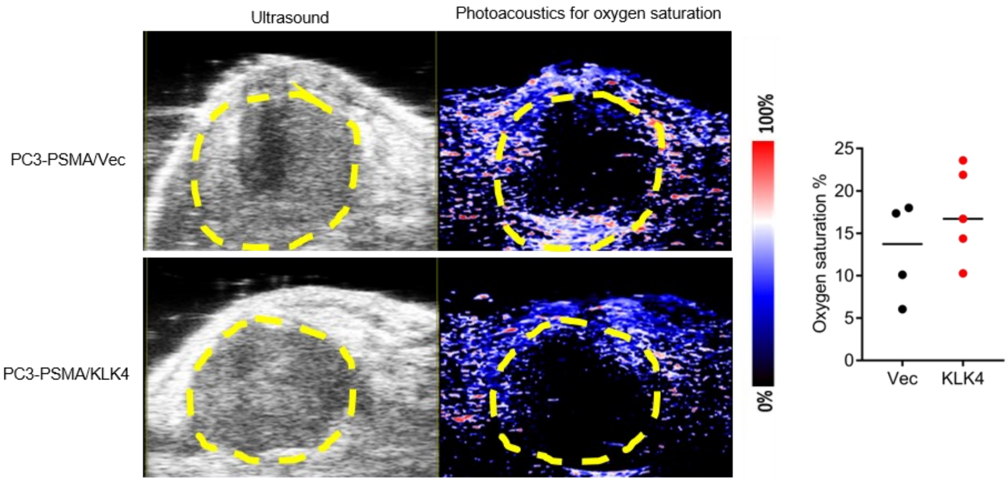

Figure S3. Photoacoustic imaging for oxygen saturation of orthotopically implanted PC3-PSMA/Vec or /KLK4 tumors at week 3 (endpoint). Photoacoustic imaging was performed on a Vevo 2100 LAZR imaging station (Visualsonics, Toronto, Canada) using a LZ400 linear array transducer, which has a center frequency of 30MHz. Depilated mice were anaesthetised by 1.5% isoflurane inhalation (1L oxygen / min) delivered via a nose cone, kept warm on a heated stage, with respiration and heart rate monitored on ECG pads. Entire tumors were scanned with their surface 10mm below the transducer, and the PA signals were amplified 40 dB. Tissue oxygen saturation can be quantified based on differences in the absorbance spectrum between oxygenated hemoglobin (Hb_{oxy}) and deoxygenated hemoglobin (Hb_{deoxy}). For oxygen saturation measurements, dual-wavelength photoacoustic imaging at 750nm and 850nm was performed. The relative amounts of Hb_{oxy} and Hb_{deoxy} were calculated using the OxyZated Tool within Vevolab software (Visualsonics, Toronto, Canada). Oxygen saturation was defined as $[100\% \times Hb_{oxy}] / [Hb_{oxy} + Hb_{deoxy}]$. The threshold for Hb was set at 70% of maximal intensity, a reasonable value determined empirically.

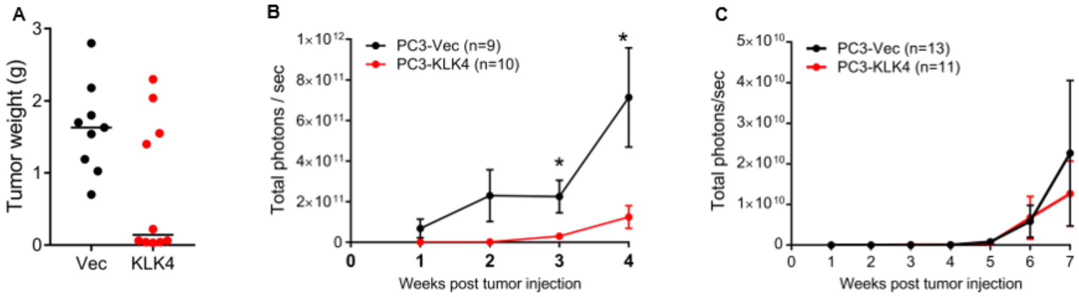

Figure S4. In vivo characterization of PC3-Vec and PC3-KLK4 cells injected intraprostatic and intracardiac in mice. **A)** Scatter plot of post-mortem weights of orthotopic PC3-Vec and -KLK4 tumors at week 4; horizontal line indicates median value. **B**) Mean tumor bioluminescence ± SEM from mice injected orthotopically with tumor cells over four time points; statistics by unpaired T-test. **C**) Mean tumor bioluminescence ± SEM from mice injected intracardiac with tumor cells over seven weeks. All presented figures are from pooled data from two independent experiments.

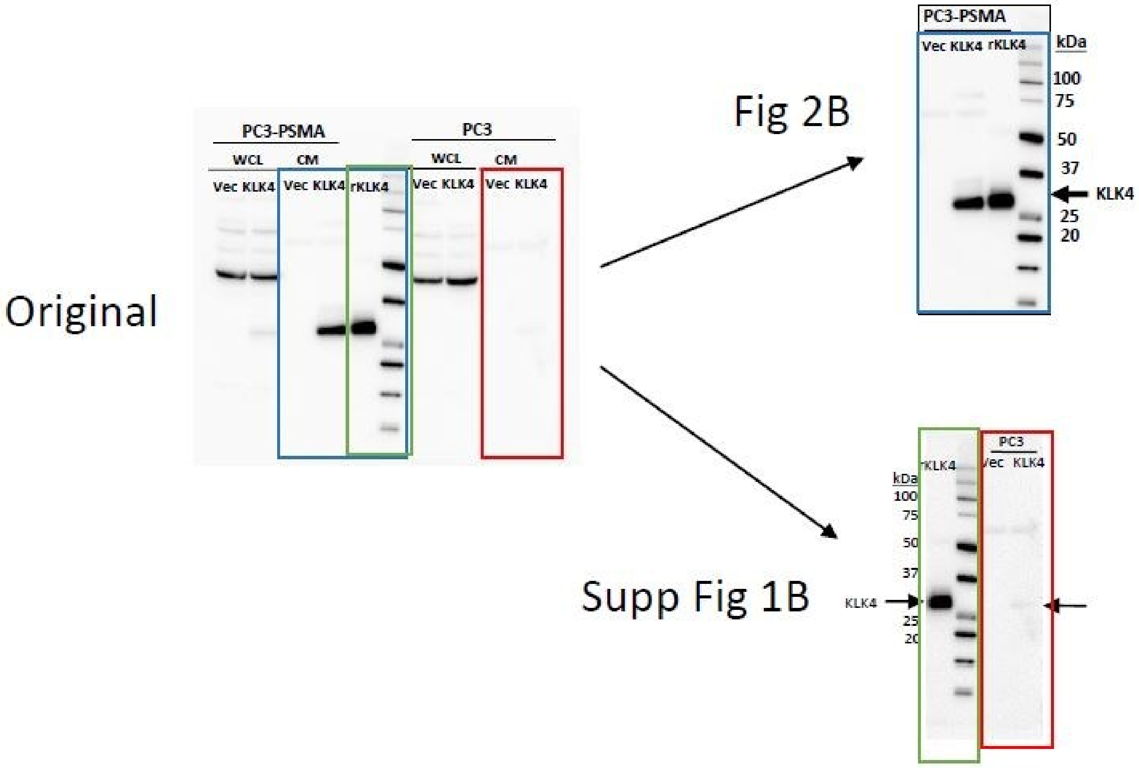

Figure S5. Uncropped Western Blot image.
